# The puzzle of self-reported weight gain in a month of fasting (Ramadan) among a cohort of Saudi families in Jeddah, Western Saudi Arabia

**DOI:** 10.1186/1475-2891-10-84

**Published:** 2011-08-10

**Authors:** Balkees Abed Bakhotmah

**Affiliations:** 1 Department of Nutrition & Food Sciences, College of Home Economy, King Abdulaziz University, P. O. Box 53100, Jeddah 21583, Saudi Arabia

**Keywords:** Fasting, Weight changes, Ramadan, Muslims, Saudi Arabia

## Abstract

**Background:**

During Ramadan fast, approximately one billion Muslims abstain from food and fluid between the hours of sunrise to sunset, and usually eat a large meal after sunset and another meal before sunrise. Many studies reported good health-related outcomes of fasting including weight loss. The objective of this study is to identify the local pattern of expenditure on food consumption, dietary habits during Ramadan and correlate that to self-reported weight gain after Ramadan in a group of families in Jeddah, Western Saudi Arabia.

**Methods:**

A Cross-section study using a pre-designed questionnaire to identify the local pattern of expenditure on food consumption, dietary habits during Ramadan and correlate that to self-reported weight gain after Ramadan in a representative cohort of Saudis living in Jeddah. It was piloted on 173 nutrition students and administered by them to their families.

**Results:**

A total of 173 Saudi families were interviewed. One out of 5 indicated that their expenditure increases during Ramadan. Approximately two thirds of the respondents (59.5%) reported weight gain after Ramadan. When asked about their perspective explanations for that: 40% attributed that to types of foods being rich in fat and carbohydrates particularly date in (Sunset meal) 97.7% and rice in (Dawn meal) 80.9%. One third (31.2%) indicated that it was due to relative lack of physical exercise in Ramadan and 14.5% referred that to increase in food consumption. Two thirds (65.2%) of those with increased expenditure reported weight gain.

**Conclusion:**

Surprisingly weight gain and not weight loss was reported after Ramadan by Saudis which indicates timely needed life-style and dietary modification programs for a population which reports one of the highest prevalence rates of diabetes.

## Background

At least, one billion of the total Muslims' population which amounts to 1.5 billion [1]. on earth refrain from eating or drinking from sunrise (Sohor)to sunset (Ifttar)during the holy month of Ramadan [2]. Ramadan is the ninth lunar month of the Islamic calendar and it will meet month of August in 2011. The fast periods in Ramadan varies from country to country and from season to season with an average length of 12 hours [2].

In Ramadan all Muslims -except children, elderly, travelers, sick and/or unable- are expected to abstain from food and drink from early dawn to sunset [3]. Among disabled individuals with acute or chronic diseases, most diabetic patients preferred to fast but certain diabetics can be exempted from fasting [4].

Although religious fasting is often a time of great spiritual growth, it can also be a time of great improvement to one's physical health and perhaps to lose weight. Most kinds of different religious fasts, and not only Ramadan fast, have this potential as forms of dietary modification [2]. During Ramadan most Muslims change their life style [3], sleep hours [5], physical activities [6], food consumption, meals frequencies and dietary habits for different reasons [2,7-10].

Fasting has been the subject of numerous scientific investigations [2,7] and [8]. The general opinion is that fasting has a potential non-pharmacological intervention for improving health and increasing longevity [7]. There are no adverse effects of Ramadan fasting on the heart, lung, liver, kidney, eyes, hematologic profile, endocrine and neuropsychiatric functions [7].

However, the majority of health-specific findings related to Ramadan fasting are mixed and sometimes contradicting [3]. The likely causes for these heterogeneous findings are the differences between studies in the following: 1) the amount of daily fasting time; 2) the percentage of subjects who smoke, take oral medications, and/or receive intravenous fluids; and 3) the subjects' typical food choices and eating habits [2]. For the last reason this study was conducted to find out the perceptions of a cohort of Saudi females and their families about their expenditure on foods during Ramadan, changes in life style, dietary behaviour/habits, meals frequencies, foods preferences, preparation of foods and its relation to body weight in view of the published literature which indicate that body mass index (BMI) may or may not decrease in response to Ramadan fasting [11-14]. It is assumed that such variation may be related to quality and quantity of foods ingested by Muslims in various countries and sub-cultures [15].

Cultures and sub-cultures differ in their socio-economic backgrounds and dietary habits in Ramadan. Most of studies published were conducted on small group of young volunteers and aimed to find out the bio-chemical, anthropometric and physiological changes under standardized strict conditions and did not approach it at a public level from all aspects including the previously mentioned factors. Understanding of the previous patterns in Ramadan will hopefully lead to better health promotion, behavior and nutrition modification programs among various communities particularly those with high prevalence rates of obesity-related type 2 diabetes such as Saudi Arabia.

## Methods

This is a cross sectional descriptive study which was performed on a cohort of Saudi females nutrition students and their families living in Jeddah city, Western Saudi Arabia. It was hypothesized that body weight will increase after Ramadan as a result of changes in life style by Saudi families which include increase in sugary and fatty foods consumption, increase in meals frequency and decrease in physical activities. In Ramadan, most Saudis have 2 main meals (Sohor before dawn and Ifttar after sunset prayers) and another 3 smaller ones (at sun-set, after night prayers i.e. Taraweh and at mid-night before Sohor).

The sample was a convenient sample. The studied group was primarily a group of 173 final year undergraduate nutrition female students who were invited to participate in filling answers to a pre-designed questionnaire. The study was conducted on Ramadan of 2008 (1429H). Students were requested to involve their parents. In case of death of both or one of parents, two mature members of the family above 20 years of age can be involved in filling the answers related to whole family food consumption pattern and to verify the accuracy of data given. Verbal informed consents were obtained after the study had been explained to them in native language. Students were living across Jeddah's city in almost all the districts. This wide comprehensive distribution of the studied clusters enhanced the representation of all the socio-economic groups of Greater Jeddah's communities which embraces more than 2.5 million inhabitants. Training and overall supervision of the interviewers were carried out by the author (BB).

For the purpose of study weight gain was simply defined as a gain of more than 3 kilograms after completing a full month of Ramadan fasting by healthy participants who were female residents in Jeddah city i.e. sick and/or travelers were excluded. In addition, non-Saudi, those who do not weigh themselves regularly or those who declined to participate were also excluded. Questionnaire was explained and administered face-to-face to nutrition students by the author. Students were also given instructions on how to fill the remaining parts of questionnaire which needs their families' assistance. The study was approved by the ethical committee of King Abdulaziz University Hospital.

A self-administered questionnaire was designed by the author based on previous experiences and knowledge of local culture to find out the perceptions of students, parents or designated members of the family about their changes in expenditure on foods, life style, meals frequency, dietary habits, food consumption and preferences, during Ramadan; and their perceptions of its relation to body weight. The questionnaire was piloted on a group of students prior to its administration.

The questionnaire consisted of six sections: (1) The socio-demographic data of the participating families which included number and gender of families members, education level of parents, monthly income (in Saudi Riyals SAR; USD = 3.75 SAR), place of residency and housing; (2) Families perspectives about expenditure during Ramadan which included: Knowledge and interest of the respondent families regarding rationalizing consumption, reasons behind increased expenditure during Ramadan; (3) Preferable timing and frequencies of meals during Ramadan; (4) Food items usually consumed during breakfast meal (Ifttar), meal's preparation and how to deal with remaining foods; (5) Food items usually consumed during Sohor meal in Ramadan and its preparation; (6) The last section was about self-reported weight gain and perspectives of studied families on underlying reasons and its relation to expenditure during Ramadan.

### Data entry and analysis

Data entry and statistical analyses were done using SPSS 16.0 statistical software package. Quality control was done at the stages of coding and data entry. Data were presented using descriptive statistics in the form of frequencies and percentages for qualitative variables, and means and standard deviations for quantitative variables. Quantitative continuous data were compared using Student t-test in case of comparisons between two groups. When normal distribution of the data could not be assumed, the non-parametric Kruskal-Wallis or Mann-Whitney tests were used instead of Student t-test. Qualitative variables were compared using chi-square test. Whenever the expected values in one or more of the cells in a 2 × 2 tables was less than 5, Fisher exact test was used instead. Pearson correlation analysis was used for assessment of the inter-relationships among quantitative variables. Statistical significance was considered at p-value < 0.05.

## Results

A total of 173 students were requested to respond to pre-designed self administered questionnaire. Table 1 describes the socio-demographic characteristics of the families enrolled in the study. Majority of families (83.8%) were constituted of six of more members with a mean ± SD accounted for 7.5 ± 2.9 members, and it was also noted that the number of males (mean ± SD 2.9 ± 1.3) was less than females (mean ± SD 3.7 ± 1.7). A slightly more than one third of the fathers (39.3%) and less percentage of the mothers (34.1%) had university qualifications. The results showed that only few minorities of the families (4.8%) had monthly income less than SAR 3000, while 41.7% had monthly income SAR 10000+. Half of the respondents (52%) were living in the North of Jeddah. Regarding the housings, half of the families (52%) were living in apartments and 40.5% were living in Villas and the rest (7.5%) were living in public houses. Almost two thirds (68.2%) of the respondents owned their houses.

**Table 1:** Characteristics of the families included in the study (n = 173)

Characteristics of the families	**No**.	%
Number of family members:		

Less than 6 members	19	11.0

6+ members	145	83.8

Missing	9	5.2

Mean ± SD	7.5 ± 2.9	

Range	2-19 members	

Number of males in the families:		

Mean ± SD	2.9 ± 1.3	

Range	1-8 males	

Number of females in the families:		

Mean ± SD	3.7 ± 1.7	

Range	1-10 females	

Education level of the father:		

Less than university	103	59.5

University qualification	68	39.3

Missing	2	1.2

Education level of the mother:		

Less than university	111	64.2

University qualification	59	34.1

Missing	3	1.7

Monthly income (in SAR)		

< 3000	8	4.6

3000- < 6000	44	25.4

6000 - < 10000	46	26.6

10000+	72	41.6

Missing	3	1.7

Place of residence		

North of Jeddah	90	52.0

South of Jeddah	38	22.0

West of Jeddah	7	4.0

East of Jeddah	26	15.0

Missing	12	6.9

Type of housing:		

Villa	70	40.5

Apartment	90	52.0

Public house	13	7.5

Ownership of the housing:		

Owned	118	68.2

Rented	52	30.1

Missing	3	1.7

**Figure 1:**
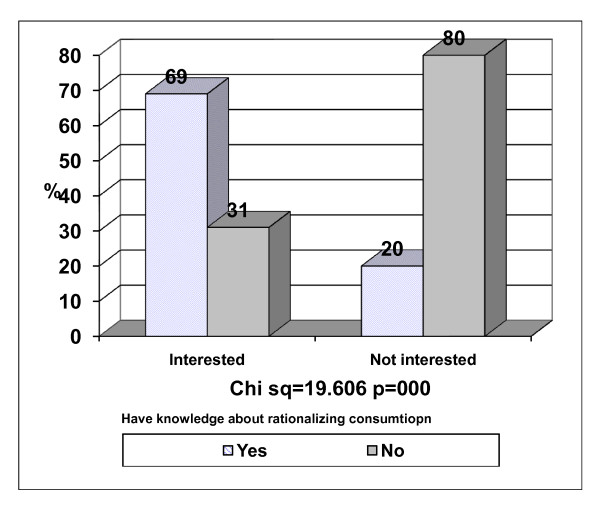
**Knowledge and interest of the respondent families regarding rationalizing consumption**.

Figure 1 displays distribution of the studied families according to their knowledge and interest about rationalizing consumption. Almost one third of the families who indicated that they had interest in rationalizing consumption (31%) had the perception of not having adequate knowledge about it, and it was observed that 20% of the families who indicated that they had enough knowledge about rationalization of consumption were not interested in adopting it. This gap in the interest and knowledge about rationalization of consumption was statistically significant p < 0.05.

Majority of the study group (79.4%) indicated that their expenditure increases during Ramadan (Table 2). This notion applies to both the families who are interested in rationalizing consumption (79.5%) and those who are not (80%). Nevertheless, it was observed that the percentage was significantly lower among families who indicated that they have adequate knowledge about rationalizing consumption (74.8%) when compared to those who did not have adequate knowledge (86.2%) p < 0.05. On the other hand it was observed that neither the monthly income nor the education level of the parents had an impact on the mode of expenditure during Ramadan p > 0.05 (Table 2). Regarding the extra expenditure of money during Ramadan, the results showed that half of those who increased expenditure during Ramadan (50.8%) are increasing it with a percentage of 50% and the other half 49.2% reported an increasing expenditure by 25%.

**Table 2:** Expenditure during Ramadan according to characteristics of the study group

		Expenditure			
				
Characteristics		Increased	Not increased	Total	p*
Family interested in rationalizing consumption	Yes	116(79.5%)	30(20.5%)	146(100%)	0.595
		
	No	20(80.0%)	5(20.0%)	25(100%)	

Family knowledgeable about rationalizing consumption	Yes	77(74.8%)	26(25.2%)	103(100%)	0.047
		
	No	56(86.2%)	9(13.8%)	65(100.0%)	

	< 3000	8(100.0%)	--	8(100%)	
		
Monthly income in SAR	3000- < 6000	33(75.0%)	11(25.0%)	44(100%)	
		
	6000- < 1000	38(82.6%)	8(17.4%)	46(100%)	0.391
		
	10000+	56(77.8%)	16(22.2%)	72(100%)	

Education level of the father	< university	83(80.6%)	20(19.4%)	103(100%)	0.595
		
	University	53(77.9%)	15(22.1%)	68(100%)	

Education level of the mother	< university	88(79.3%)	23(20.7%)	111(100%)	0.561
		
	University	47(79.7%)	12(20.3%)	59(100%)	

Total		135(79.4%)	35(20.6%)	170(100%)	

**Figure 2:**
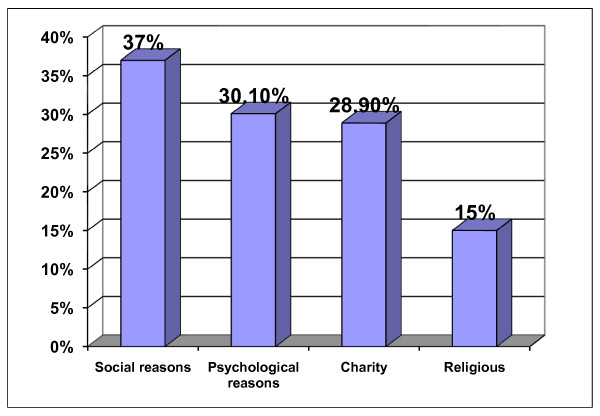
**Reasons behind increasing expenditure during Ramadan**.

The reasons behind increasing expenditure during Ramadan were displayed in Figure 2 in descending order. The social reasons including increased frequency of family gatherings, invitations and celebrations constituted the main reason (37%), followed by the psychological reasons (30.1%) in the form of the insight desire for imitating others. The charity and religious factors came after as reasons for increasing expenditure during Ramadan (28.9) and (15%) respectively.

**Figure 3:**
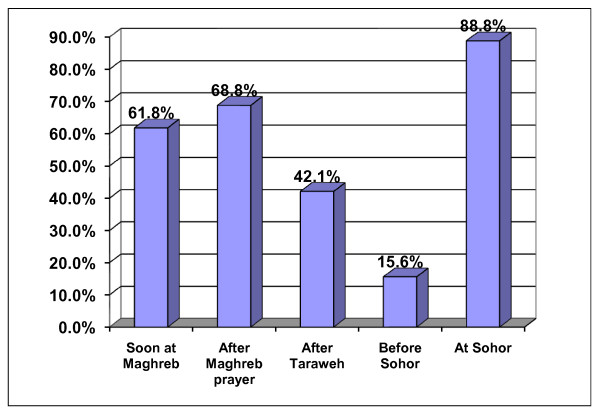
**Preferable time for taking meals during Ramadan**.

Regarding timing and frequencies of meals during Ramadan, the majority of the respondents (88.8%) pointed out that they take a main meal at Sohor, and slightly more than two thirds (68.8%) indicated that they take a meal immediately after sunset i.e. Maghreb prayer (Figure 3).

**Figure 4:**
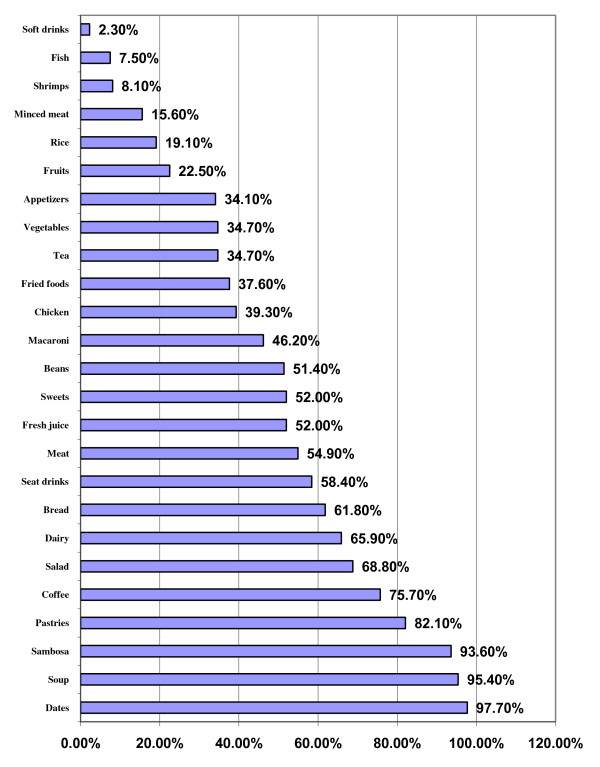
**Food items usually consumed during breakfast meal (Ifttar) of Ramadan**.

In Figure 4 the foods commonly consumed at Ifttar are displayed. Dates are on the top of list (97.7%) at breakfast meal (Ifttar) during Ramadan, followed by meat soups (95.4%) and Sambosa or Samosa which is pastry filled with meat or cheese (93.6%). Also, it was noted that the majority of the respondents (82.1%) are consuming pastries and coffee (75.7%). Almost two thirds of the respondents (68.8%) indicated that they consume salads, dairy products (65.9%) and bread (61.8%). On the other hand, it was observed that the least food items to be consumed during Ramadan are the fisheries, where it was found that only 8.1% of the respondents indicated that they eat shrimps regularly and 7.5% marked that they consume fish. Soft drinks were used by very few minorities of the families (2.3%), (figure 4).

Table 3 illustrates the breakfast meal preparation, consumption and the ways of dealing with the remnants of food stuffs. Majority of families (75.1%) were rarely buying readymade foods for breakfast and only (1.8%) always buy it from outside. Meanwhile, it was found that the father is the chief person (56.1%) responsible for buying the food ingredients, and the mother is the main person shouldering the responsibility of preparing the breakfast meal (74.6%), and 12.1% of the families were depending on the maids for preparing it. Regarding the breakfast meals' remnants, it was realized that in general 84.4% of the families indicated that they usually have remnants of the breakfast meals. One third of the families indicated that always there are remnants of the breakfast meals in addition to 51.5% who indicated that it is sometimes occurring. Moreover, it was realized that the majority of these remnants (75.2%) are stored for being used later, and 22.5% are using it for charity purposes. Only few minorities (2.3%) addressed that they are discarding the remnants of breakfast meals (Table 3).

**Table 3:** Breakfast meal preparation and consumption during Ramadan

	No	%
Buying prepared food from outside		

Always	3	1.8

Sometimes	40	23.1

Rarely	130	75.1

The person who is buying food ingredients		

Father	97	56.1

Other family member	43	24.9

Mother	33	19.0

The person who is preparing breakfast meals		

Mother	129	74.6

Maid	21	12.1

Other family members	23	13.3

Are there remnants of the breakfast meals?		

Always	57	32.9

Sometimes	89	51.5

Rarely	27	15.6

Dealing with remnants of the breakfast meals		

Charity for poor	39	22.5

Stored for being used later	130	75.2

Discarded	4	2.3

**Figure 5:**
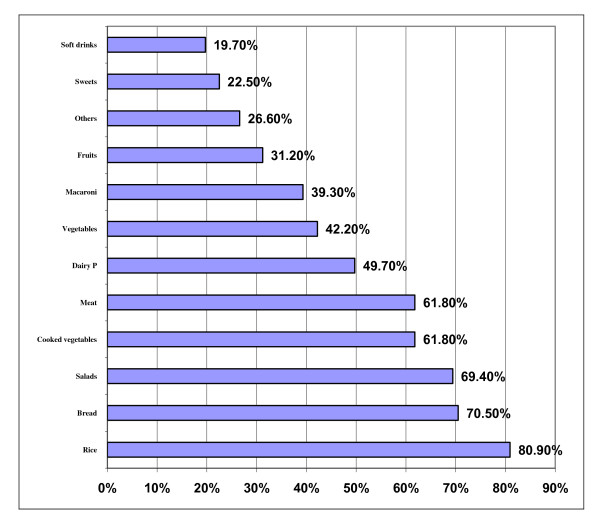
**Food items usually consumed during Sohor meal in Ramadan**.

Rice headed the food items usually consumed in Sohor during Ramadan (80.9%) followed by bread (70.5%) and salads (69.4%), (Figure 5). At the same time, it was remarked that almost two thirds of the families are taking meat and cooked vegetables (61.8%) in Sohor. In addition, half of the families (49.7%) are consuming dairy products and slightly less than half of them are eating fresh vegetables. On the other side, it was realized that the least food items consumed by the families in Sohor were the soft drinks (19.7%) and sweets (22.5%).

In contrast to breakfast meal where the respondents prepare it at home, (59%) of the families are buying prepared food for the Sohor meal, and more than one third (38.7%) are doing so sometimes. Only few minorities (2.3%) indicated that they are always buying readymade food for Sohor meals.

Almost two thirds of the respondents (59.5%) self-reported weight gain among some or all of the family members after Ramadan, and when asked about their perspective explanation for the weight gain, 40% attributed the weight gain to the types and quality of foods usually consumed during Ramadan being rich in fat and sweets, and almost one third (31.2%) articulated that the reason for weight gain during Ramadan is the lack of physical exercise (Table 4).

**Table 4:** Self-reported weight gain during Ramadan

	No	%
Remarked weight gain in some or all of the family members after Ramadan		

Yes	103	59.5

No	70	40.5

Reasons of weight gain		

Consumption of extra food	25	14.5

Type of food (fatty and sweets)	70	40.5

Lack of physical exercise	54	31.2

Others	24	13.8

It was obvious that two thirds (65.2%) of those who indicated that there is increased expenditure during Ramadan realized weight gain among some or all of family members after Ramadan. The reverse was observed for those who indicated that they did not increase expenditure during Ramadan as they expressed no weight gain realized among their family members (62.9%) and this difference was statistically significant p < 0.05.

## Discussion

Ramadan fasting is a religious obligation. Therefore no randomized controlled trial was done to ascertain its benefits, and data available in literature relies on before-after studies^(8)^. Most of studies published are on young individuals, conducted in heterogeneous communities of diverse cultural back grounds and diverse dietary habits [2,8-13]. No large-scale studies have yet been done in Middle East and North African MENA countries including Saudi Arabia [8]. Those countries are witnessing an "epidemic" of type 2 diabetes mellitus which is strongly related to obesity [16]. For those countries, Ramadan fasting is theoretically a golden opportunity to adopt healthy life style and dietary habits which will lead to weight reduction, better control of diabetes and its complications and perhaps other biochemical changes associated with metabolic syndrome [17-19].

This pilot community-based study aimed to look at weight changes and its relation to different variables related to food consumption, dietary habits and behavior as reported by a representative group of Saudi families living in Jeddah, Saudi Arabia. The sample of studied families was distributed across Jeddah and represented all of the socio-economic groups of Jeddah's community Table 1.

Most of Muslims' families believe that Ramadan is a month of "giving" and generosity. Therefore it is not surprising that there is a gap between knowledge and interest in rationalizing consumption and expenditure Figure 1. This gap should be bridged by more behavior/nutrition education programs especially designed to our local community about the concept mentioned above. The nutrition educationalists and perhaps social/religious leaders should be involved in filling the gap. This was supported by the finding in Table 2 which showed that knowledge has a significant impact on rationalizing expenditures. Similar observation of increase food consumption in Ramadan was noticed in Algerian study [4] and Moroccans youngsters living in Spain [2].

Social reasons headed the lists of reasons behind increasing expenditure during Ramadan followed by psychological reasons, charity and finally religion. This indicates the importance of changing the above behaviors to healthy behaviors which include education of local community that social gathering should not be always accompanied by more food intake.

Unlike other Muslims in MENA countries [2,12,19], the studied Saudi families increased the frequency of meals from 3 outside Ramadan to 5 in Ramadan Figure 3. Unfortunately, the "Sohor" meal is one on the top. This particular meal is always followed by late sleeping for at least 5 hours. In Tunisia, the main meal is breakfast and not "Sohor" [20].

As noticed by other authors in previous studies in various Muslim's countries most Muslims usually increase food consumption particularly proteins and fat but not carbohydrates [4,9,20] as observed in Saudis. Almost all of Saudis have dates at breakfast time (97.7%), followed by soup and Sambosa (93.6%). The dates are too sugary and the Sambosa is fatty as it is usually fried and filled with minced meat. Soup is usually prepared using meat and chicken, Figure 4.

Breakfast meal is rarely bought from outside (1.7%). This is a healthy behavior and should be supported assuming that the family will change the bad cooking habit like frying and avoid the fatty and sweaty items. A similar trend was reported by Guerrero Morilla et al. [9]. In Table 3, 32.9% of families reported that there is always remaining food. This indicates again the need for more efforts on educating the local community to avoid over spending in Ramadan.

In contrast to breakfast meal, Sohor meal which is the main meal contains rice as a main food almost 80% of responding families. It is commonly cooked using meat. The most traditional dish in Saudi Arabia is "Kabsah" which is rice plus meat. It is of high calories and fat contents. "Kabsah" is commonly bought from fast-foods traditional shops and not cooked at home. In Ramadan less vegetable and fruits were consumed in both breakfast and "Sohor" meals.

These practices and dietary habits of Saudis are different from other MENA countries such as Tunisia [20], Algeria [21], Egypt [19], Jordan [11], Turkey [18] and Iran [7]. This variation in food expenditure, food consumption, increase meals frequency, the bad food preferences may explain the contradicting finding in this study compared to the previously mentioned studies in which weight loss and not weight gain was reported. It may not be a surprise therefore that 59.5% of the sample reported weight gain in Ramadan rather than no change or weight loss. Unfortunately, only half of them correlated that weight gain to the consumption of extra-food and the choice of fatty and sugary foods. This supports the previous findings of studies conducted on Saudis two decades ago which indicated that Saudi Muslims increase their energy intake compared to Indians Muslims [2,7] and [15].

In view of the findings of this study, it is believed that weight gain and not weight loss is the problem in Saudi Arabia which is a developing Muslim country with high prevalence rates of diabetes mellitus which amounted to 23.7% as reported by Al Nozha et al. [22] mainly Type II which is strongly related to obesity. On the long term the current dietary habits are not healthy to the Saudi community particularly diabetics and may contribute to higher prevalence rates of diabetes-related complications on long term. Theoretically, Ramadan fast model is a good model for behavior modification [23] and health promotion. It is a good opportunity for not only maintaining weight but also to reduce it in obese and overweight individuals [4,13,18,19]. In Jeddah, Saudi Arabia these goals are not met for many reasons described above. The gap between knowledge and practice should be filled by more nutrition education about reducing the numbers of meals, improving the dietary habits, improving the quality and quantity of food intake, and increase instead of current decline in physical activity. Education awareness campaigns may not succeed unless it is accompanied by policies which enforce the media to reduce the amount of foods advertisement about foods. Social researchers should work on changing the concept of many Muslims that Ramadan is a month of having more foods at night to compensate for the fasting periods. Diet in Ramadan should not differ very much from a healthy normal diet which maintains normal weight and if one is over-weight, Ramadan is a good time to shed some pounds. More research is needed in Saudi Arabia and adjacent Gulf Countries GCC to study the observation of changing the fasting month of Ramadan from a month of fasting to an "over-eating" 's month.

## Conclusion

In contradiction to what is logically expected after a month of fasting and to what was reported in literature, it is found in this study that weight gain and not weight loss was likely to happen after Ramadan by Saudis living in Jeddah. The findings in this small-scale cross-sectional study indicates the need for larger scales studies at national levels not only in Saudi Arabia but also in adjacent gulf countries and perhaps MENA countries of similar cultural and social back grounds. There is need for educational programs which focus on healthier life-style in Ramadan and dietary modification of a population which reports one of the highest prevalence rates of diabetes.

## Abbreviations

Ramadan: The holy month of fasting; Ifttar: the breakfast meal at the sunset; Sohor: the dawn meal at late night; MENA: Middle East and North African countries; Maghreb prayer: sunset prayer; Taraweh prayer: mid-night prayer.

## Competing interests

The author declares that they have no competing interests.

## Authors' contributions

The author has contributed to design, conduct and preparation of the final version of this manuscript.
